# Global rigid registration of CT to video in laparoscopic liver surgery

**DOI:** 10.1007/s11548-018-1781-z

**Published:** 2018-05-07

**Authors:** Maria R. Robu, João Ramalhinho, Stephen Thompson, Kurinchi Gurusamy, Brian Davidson, David Hawkes, Danail Stoyanov, Matthew J. Clarkson

**Affiliations:** 1 Wellcome/EPSRC Centre for Interventional and Surgical Sciences, University College London, London, UK; 2 Centre For Medical Image Computing, University College London, London, UK; 3 Division of Surgery and Interventional Science, University College London, London, UK

**Keywords:** Image guidance, Laparoscopic liver surgery, Global registration, Shape matching, Surface descriptors, Computer-assisted surgery

## Abstract

**Purpose:**

Image-guidance systems have the potential to aid in laparoscopic interventions by providing sub-surface structure information and tumour localisation. The registration of a preoperative 3D image with the intraoperative laparoscopic video feed is an important component of image guidance, which should be fast, robust and cause minimal disruption to the surgical procedure. Most methods for rigid and non-rigid registration require a good initial alignment. However, in most research systems for abdominal surgery, the user has to manually rotate and translate the models, which is usually difficult to perform quickly and intuitively.

**Methods:**

We propose a fast, global method for the initial rigid alignment between a 3D mesh derived from a preoperative CT of the liver and a surface reconstruction of the intraoperative scene. We formulate the shape matching problem as a quadratic assignment problem which minimises the dissimilarity between feature descriptors while enforcing geometrical consistency between all the feature points. We incorporate a novel constraint based on the liver contours which deals specifically with the challenges introduced by laparoscopic data.

**Results:**

We validate our proposed method on synthetic data, on a liver phantom and on retrospective clinical data acquired during a laparoscopic liver resection. We show robustness over reduced partial size and increasing levels of deformation. Our results on the phantom and on the real data show good initial alignment, which can successfully converge to the correct position using fine alignment techniques. Furthermore, since we can pre-process the CT scan before surgery, the proposed method runs faster than current algorithms.

**Conclusion:**

The proposed shape matching method can provide a fast, global initial registration, which can be further refined by fine alignment methods. This approach will lead to a more usable and intuitive image-guidance system for laparoscopic liver surgery.

## Introduction

Minimally invasive surgery offers the patient major benefits over open surgery, including less trauma, less pain and shorter hospital stays. However, these interventions present several challenges for clinicians, such as weak depth perception, constrained vantage point, limited field of view, poor haptic feedback and occluded anatomy [1]. Image guidance aims to assist the clinicians in localising and tracking sub-surface structures such as abnormalities or major vessel trees. Thus, these systems have the potential to aid in surgical interventions through improved resection quality and a reduction in positive surgical margins [2]. The safety margin around a possible tumour in current laparoscopic procedures is a minimum of 10 mm [3], so it is considered desirable to develop systems with accuracy below 5 mm on average [4, 5]. Current rigid registration methods achieve accuracies of approximately 10 mm in phantom experiments [5–7]. Improving the robustness, accessibility and reliability of image-guidance systems could potentially increase the number of patients benefiting from minimally invasive surgeries.

Most hospitals require an abdominal CT scan to be acquired before surgery for laparoscopic liver interventions. A 3D model of the liver, major vessel trees and any abnormalities can be segmented from the CT scan. The registration of the preoperative liver model and the intraoperative laparoscopic images is an essential step towards developing an image-guidance system. Most methods in the literature can be divided between coarse alignment, defined as a global alignment which can match the surfaces irrespective of their initial transformation, and fine alignment techniques—in which a good initial alignment is already provided as a starting point. In this paper, we focus on coarse alignment methods for surface-based registration.

Furthermore, most methods are only applicable in open surgery [4, 6–8] as a large surface of the intraoperative scene is required. However, surfaces acquired laparoscopically present the additional challenges that the camera has access to a restricted region of the abdomen leading to an even smaller partial view, lack of reliable landmarks and significant deformation from pneumoperitoneum [7]. We address and discuss the challenges inherent to laparoscopic surgeries which motivated our method.

We propose a fast, semi-automatic global alignment method which achieves the initial alignment between the preoperative CT model of the liver surface and a surface reconstruction of the intraoperative scene. The resulting transformation could be further improved by fine alignment algorithms [9, 10] in order to get a rigid [5, 6, 11] or a non-rigid alignment [3] between the two modalities. Our approach can lead to a faster and more intuitive use of image-guidance systems in laparoscopic surgeries. We show robustness to reduced partial sizes and increasing deformations in the intraoperative model on synthetic data. Moreover, we evaluate the proposed method on a liver phantom and on retrospective data from a dataset acquired in a laparoscopic liver resection with promising results.

### Background

The initial rigid registration of the preoperative 3D image and the intraoperative scene has been explored through methods that rely on fiducials, user interaction and through fully automated methods.

Several approaches propose the use of fiducials, either on the patient skin [12] for needle guidance, or on the organ itself [13] for tracking in laparoscopic partial nephrectomy. Another more robust option, which is applicable in laparoscopic interventions, would be to attach metabolisable fluorescent markers on the organ [14]. Such fiducials can be seen in both modalities. However, these strategies are disruptive to the clinical workflow since they require the acquisition of an additional CT or MRI scan immediately before the intervention.

**Fig. 1:**
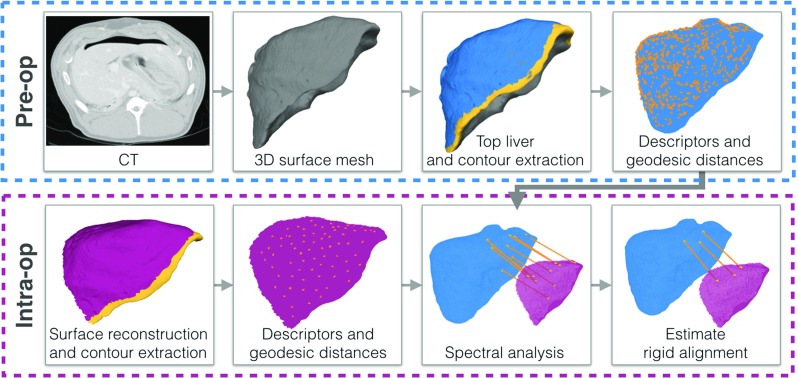
An overview of our proposed global alignment framework, showing the preoperative and intraoperative steps

Surface acquisition of the intraoperative scene has been proposed as an alternative. Several strategies have been developed using laser range scanners [6], optically tracked probes [11], time-of-flight (TOF) data [7] and stereo reconstruction [5]. Once the surface is acquired, the clinician is required to delineate salient anatomical features leading to a point-based initial alignment [6, 11] or to a more complex non-rigid optimisation framework [3]. Another option to obtain the rigid alignment is to manually rotate and translate the 3D preoperative image until it fits the intraoperative data [5]. While some level of user interaction is needed for these approaches, it is generally more intuitive and faster to select salient features than to manipulate the six degrees of freedom associated with a rigid transform.

Hybrid methods have been proposed using cone beam CT and fluoroscopy [15] as bridging modalities between the laparoscopic camera and the preoperative CT, which delivers an additional radiation dose to the patient. Feuerstein et al. [16] propose using intraoperative cone beam CT and optical tracking to register directly to the laparoscopic view without using preoperative information. While their methods achieve promising results, they are based on advanced hardware which might not be available in most clinical settings.

Finally, fully automated techniques have been proposed in [7, 17]. Fusaglia et al. [17] developed an exhaustive search over the principal directions of the intraoperative surface, which is acquired using a laparoscopic laser pointer. While their proposed approach is promising, it still introduces additional tools into the clinical workflow. Dos Santos et al. [7] introduced a novel automatic method to establish surface correspondences between the 3D preoperative mesh and the intraoperative surface acquired with a TOF camera in open liver surgery. Their approach was validated on a phantom of the human liver and on an ex-vivo porcine liver with accuracy better than 1 cm and computation time ranging from one minute to 5.5 h. While their phantom validation under deformation from breathing motion can be sufficient for open surgery, livers in laparoscopic interventions undergo significant general deformation due to pneumoperitoneum. Furthermore, it is unclear how both methods [7, 17] would be translated to laparoscopic interventions since they rely on large surfaces of the liver being visible.

While promising results have been achieved in the literature, we aim to develop an image-guidance system which can handle the challenges of laparoscopic interventions and is easy to integrate with the current clinical protocol without additional hardware or advanced cameras. Furthermore, the system should be usable during surgical interventions, with minimal disruption and fast computation.

### Contributions

We propose a fast, semi-automatic method to obtain a global initial alignment between a 3D liver model extracted from the preoperative CT scan and a surface reconstruction of the intraoperative scene.

An existing formulation of shape matching is extended to incorporate an additional constraint based on the contours of the organ (the ridge line—see Fig. 1), which can be identified on both surfaces with high confidence. Once the delineation of the liver ridge line is given in the two modalities, no further user interaction or initialisation is required for the alignment stage. The proposed method is able to robustly estimate a correspondence set between the two surfaces under deformation, sparse data, partial views and realistic noise levels.

We validate our technique in a simulated environment to show robustness to partial data and deformation. Moreover, we provide quantitative results obtained on a liver phantom and qualitative results on retrospective data from a laparoscopic liver resection to illustrate feasibility in a realistic clinical setting.

## Methods

Figure 1 illustrates the main steps of the proposed pipeline. The input data include the segmentation of the 3D mesh from the CT scan, a surface reconstruction represented as a point cloud of the intraoperative data and the segmented contours on both surfaces. The liver contour is defined as the ridge line visible in yellow in Fig. 1 on both the preoperative and the intraoperative surfaces.

Let *M* be the moving (preoperative CT mesh) model, and let *T* be the target (intraoperative point cloud) model. Sets of features, $\{m_r\} \subset M$ and $\{t_s\} \subset T$, are selected on both surfaces with $f(\cdot )$ as their corresponding descriptor. Let $d_g(x,y)$ be the geodesic distance between any two points, *x* and *y*, on a surface.

Generally, it is difficult to match surfaces in laparoscopic liver surgery only based on descriptors since the surfaces lack prominent, uniquely identifiable features. The use of geometric consistency between the correspondences on both shapes can further constrain the registration problem.

Shape matching can be formulated as a quadratic assignment problem (QAP):

1 $$\begin{aligned} E(C)= & {} \sum _{(m,t)_i \in C} d(f(m_i),f(t_i)) \nonumber \\&+ \sum _{(m,t)_i \in C} \sum _{(m,t)_j \in C} (d_g(m_i,m_j) - d_g(t_i,t_j))^2 \end{aligned}$$

where  is the initial correspondence set composed of candidate pairs of feature points from the two surfaces, $d(f(\cdot ),f(\cdot ))$ is the distance between the feature descriptors and $d_g(\cdot ,\cdot )$ is the geodesic distance between two correspondences on the same surface. This energy function aims to output a set of correspondences for which the dissimilarity between the descriptors is minimised and the geodesic distances between pairs of correspondences are maintained.

**Fig. 2:**
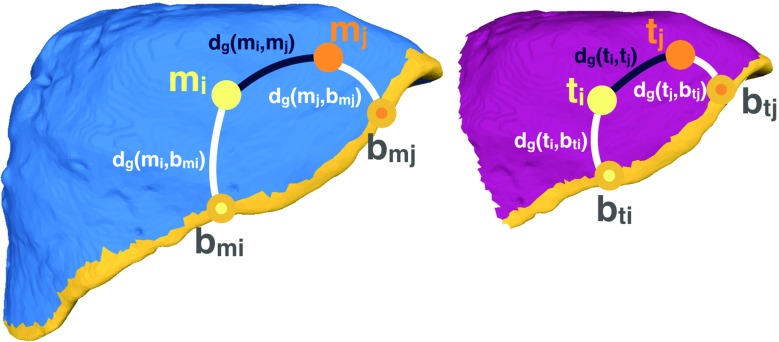
Pairwise constraints on the moving, *M* (blue) and target, *T* (pink) models used for pruning the correspondence set

While this approach works well in the vision literature for complex shapes [18], the intraoperative surfaces pose several challenges. It has been previously discussed in [7] that constraining the correspondence set based only on the geodesic distances between them is still ambiguous for almost flat surfaces, in which the same spatial configuration of features can be identified in multiple locations. The same behaviour was observed with our data. So, an additional constraint based on the liver contour is proposed, which can be reliably observed on both models. The existing spectral matching framework is extended to incorporate the new term and robustly estimate a set of correspondences.

### Optimisation

In order to minimise *E*(*C*) from Eq. 1, the shape alignment problem is formulated as graph matching [19]. Each node consists of a candidate correspondence (i.e. $(m,t)_i$), and each edge connects two nodes (i.e. $(m,t)_i$ and $(m,t)_j$). Moreover, if pair $(m,t)_i$ corresponds to $(m,t)_j$, the pairwise constraints imposed will quantify how consistent this association is from a geometrical point of view, thus providing weights for the edges. Figure 2 highlights an example of a correct assignment.

An affinity matrix, *W*, of the graph is built. The weights associated with each node and edge will result in a strongly connected cluster for data with high consistency. On the other hand, outlier nodes will be either weakly linked or linked in an unstructured way. In cases with a high number of outliers, large deformation or symmetry in the data, some wrong correspondences might be included in the main cluster. As a result, the initial correspondence set *C* is built by choosing for each target feature point $\{t_s\} \subset T$, the closest *k* neighbouring descriptors on the moving surface $\{m_r\} \subset M$, quickly using *kd trees*. A spectral analysis method [19] is used to obtain the filtered correspondence set $C_\mathrm{p}$ from the matrix *W*. In the next few paragraphs, the proposed formulation for *W* is detailed, which incorporates the additional term based on the liver contours.

The affinity matrix, *W*, should have values which are non-negative, symmetric and increasing with higher similarity between the correspondences [19]. So, instead of working with distances as in Eq. 1, the pairwise terms are parametrised as consistency measures:

2 $$\begin{aligned} c(m_i,m_j,t_i,t_j) = \hbox {min} \left[ \frac{d_g(m_i,m_j)}{d_g(t_i,t_j)+\varepsilon },\frac{d_g(t_i,t_j)}{d_g(m_i,m_j)+\varepsilon } \right] \nonumber \\ \end{aligned}$$

where $\varepsilon$ is a small number to ensure the denominators are not zero, $c \in [0,1]$ and it quantifies how similar the geodesic distances are between the pairs $(m_i,m_j)$ and $(t_i,t_j)$. A pair of correspondences is consistent from a geometric point of view if the ratio of the geodesic distances on each shape is close to 1 [20]. However, in the presence of non-isometric deformation of the data, correct correspondences might have consistency values, *c*, lower than 1. So, the non-rigidity of the data is taken into account by using the following function for *c*:

3 $$\begin{aligned} g(m_i,m_j,t_i,t_j,\sigma ) = \hbox {exp} \left( -\,\frac{(c(m_i,m_j,t_i,t_j) - 1)^2}{2 \sigma ^2} \right) \nonumber \\ \end{aligned}$$

where the parameter $\sigma$ sets the amount of non-rigidity allowed for the correspondence set. Furthermore, the function *g* also helps in separating the outliers by lowering the weights of highly unlikely candidate pairs.

Let $B^M \subset M$ and $B^T \subset T$ be the contour points on each surface. The closest contour point to each feature point *x* on either *M* or *T* is computed as $b_x = \hbox {min} (d_g(x,B))$. The expression used for the proposed contour constraint is:

4 $$\begin{aligned}&g_b(m_i,m_j,t_i,t_j,\sigma _b) \nonumber \\&\quad = \frac{1}{2} \Big ( g(m_i,b_{mi},t_i,b_{ti},\sigma _b) + g(m_j,b_{mj},t_j,b_{tj},\sigma _b) \Big )\nonumber \\ \end{aligned}$$

where $\sigma _b$ allows some deformation between the candidate pairs and their corresponding contours. In practice, $\sigma _b = \sigma _d$ since they both represent variations in geodesic distances illustrating how restrictive the geometric pairwise constraints will be on the data.

Finally, the affinity matrix, *W*, is built by placing the unary terms (similarity between descriptors $\hbox {sim}(f(m_i),f(t_i))$) on the main diagonal and the pairwise constraints on the off-diagonal:

$$\begin{aligned}&W(i,j) \\&\quad ={\left\{ \begin{array}{ll} \hbox {sim}(f(m_i),f(t_i)), i=j\\ \alpha g(m_i,m_j,t_i,t_j,\sigma _d) + (1-\alpha ) g_b(m_i,m_j,t_i,t_j,\sigma _b) , i \ne j \end{array}\right. } \end{aligned}$$

where $\alpha$ allows different weights for the importance of the two pairwise constraints.

Spectral analysis on the initial formulation for *E*(*C*) (Eq. 1) enforces high similarity between nodes $(m,t)_i$ and $(m,t)_j$, as well as approximately equal distances between them ($d_g(m_i,m_j)$ and $d_g(t_i,t_j)$). In addition, the proposed term weighs the edge connecting $(m,t)_i$ and $(m,t)_j$ higher if the distances $d_g(m_i,b_{mi}), d_g(m_j,b_{mj})$ are similar to $d_g(t_i,b_{ti}), d_g(t_j,b_{tj})$, respectively. As a result, the estimated correspondence set, $C_\mathrm{p}$, is explicitly constrained to be consistent with the liver contours on both surfaces, *M* and *T*.

### Features and descriptors

Reliable landmarks are difficult to identify consistently between the two surfaces. The strategies used are farthest point sampling [21] and normal space sampling [22]. The former approach was chosen for a uniform distribution of the feature points on the surface. The latter aims to select samples such that the normals are distributed as evenly as possible, thus having fewer points in flat regions. The search space is constrained to only select features on the visible surface of the moving mesh, *M*, in order to eliminate unfeasible solutions (see Fig. 1). TOLDI was chosen as a feature descriptor, because it was shown to be robust to data sampled irregularly (which is the case for multiple stereo reconstruction surfaces merged together), robust to varying levels of noise and invariant to rigid transformations [23].

### Distances

The geodesic distance represents the shortest distance on the surface between two points. If the surface changes topology through holes or irregularities in the data, the geodesic distances might become unreliable. Another failure case would be observed if distant parts of the object come into contact and create new shortest paths between feature points. However, it is unlikely the liver shape will change topology in the initial stages of the surgery.

The intraoperative data collected during laparoscopic surgery will most likely have some degree of sparsity—sparse point clouds [3], sparse data collection [4], sparse stereo reconstructed patches [5].

So, let *S* be a smooth interpolated surface of the intraoperative point cloud, *T*. The target feature points, $\{t_s\}$, and contour points, $B^T$, can be expressed on the interpolated surface with nearest neighbour computation. The geodesic distances on *S* are computed using the fast marching algorithm [24, 25]. This step is the most computationally expensive to compute in our implementation. However, faster alternatives can be employed [26].

### Estimating the rigid transform

The proposed shape matching technique starts with a large set of correspondences, *C*, and spectral analysis prunes out the outliers, resulting in $C_\mathrm{p}$. The final set of correspondences is not guaranteed to consist only of correct matches, especially in cases with significant deformation.

Random sampling and consensus (RANSAC) [27] (see Fig. 1) is used to get the best minimal solution $\{(m,t)_i\} \in C_\mathrm{p}$ out of the pruned set of correspondences $C_\mathrm{p}$. The final pairs $\{(m,t)_i\}$ are used for the least squares estimation of rotation and translation. The estimated transformation is considered to be a good fit if the root-mean-squared error (RMSE) between the target and moving models is less than a threshold $d_\mathrm{RANSAC}$ and the difference between the normals is less than a specific angle threshold $a_{\mathrm{normals}}$: dot$(n_m,n_t) < \hbox {cos}(a_\mathrm{normals})\quad \forall \; {(n_m,n_t)_i} \in C_\mathrm{p}$.

**Fig. 3:**
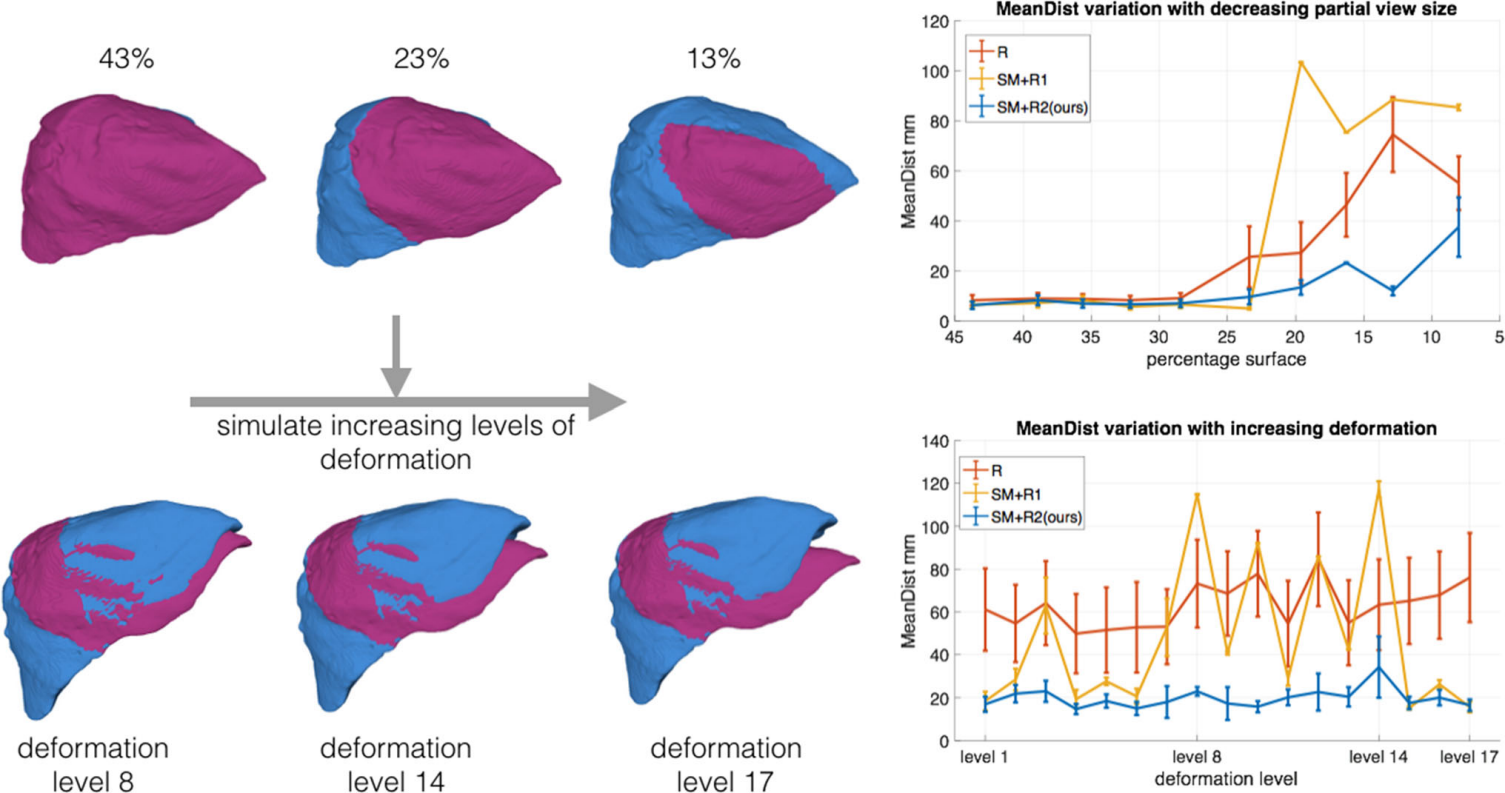
Experiments on synthetic data. Top: robustness to reduced partial size in the target model, *T*, bottom: robustness to increasing levels of deformation in *T*. The target model representing 23% of the total liver surface is used in the bottom experiment with increasing deformation levels. *Color coding:* moving model, *M*—blue, target model, *T*—pink

## Results

Three sets of experiments were conducted to validate the proposed method. Firstly, the robustness to the specific challenges present in laparoscopic interventions (partial views, varying degrees of deformation) was tested on synthetic data. Secondly, the proposed initial alignment method was quantitatively validated in a liver phantom experiment. Finally, qualitative results are shown on retrospective clinical data from a dataset acquired during a human liver resection.

The same parameters are used for all our experiments both on synthetic and on clinical data ($\sigma _d = \sigma _b = 0.3$, $\alpha = 0.6$). The maximum number of iterations used for RANSAC is 1000. The difference between the normals is set as $a_\mathrm{normals} = 60^{\circ }$ in order to account for some of the deformation. Similarly, $d_\mathrm{RANSAC} = 5\,$mm in the rigid case scenario (see “Robustness to reduced partial size” section) and $d_\mathrm{RANSAC} = 10\,$mm for the remaining experiments. If no solution is found with the RMSE lower than $d_\mathrm{RANSAC}$, the transformation which resulted in the smallest error over all the iterations is used. The feature points, descriptors and geodesic distances on the CT mesh are precomputed and stored, in order to minimise the computation time during surgery. The liver contours are currently manually delineated in a matter of seconds, and techniques to automate this step will be investigated in the future.

The proposed method was implemented in Matlab and C++, on a MacOS 10.11.2 laptop with an Intel Core i7 3.1 GHz processor. The libraries used as dependencies can be found in [21, 23, 25, 28]. The mesh processing applications MeshMixer^1^ and Meshlab [29] were used for visualisation and simulation purposes.

### Synthetic data

We validate the robustness of the proposed method to partial views of the liver and to increasing deformation levels. The mesh of a liver phantom (OpenCAS [8]) is used as the moving model, *M*. The mean distance between the estimated registration result and ground truth vertex correspondences is measured. Three algorithms are compared:

- (R) RANSAC applied directly to the initial set of correspondences, *C*. No pruning is applied.
- (SM $+$ R1) The initial set, *C*, is pruned based on geodesic distances alone, following the spectral analysis technique detailed above. RANSAC is applied on the pruned set, $C_\mathrm{p}$.
- (SM $+$ R2—ours) The proposed technique with both pairwise constraints.

**Fig. 4:**
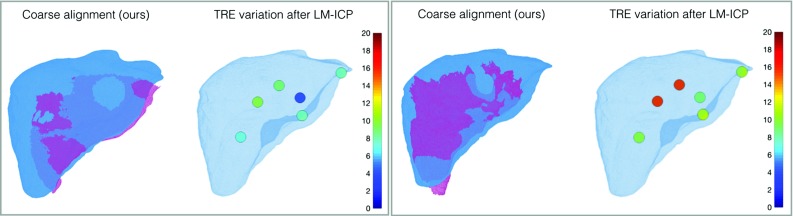
Phantom experiment. Our proposed global initial alignment is sufficient to allow potentially any fine alignment method to successfully converge. The TRE distribution after convergence of LM-ICP [10] is shown for a partial region of the deformed surface (left) and a partial surface reconstruction from an intraoperative stereo laparoscopic camera (right) *Color coding:* moving model, *M*—blue, target model, *T*—pink

#### Robustness to reduced partial size

We test the robustness of the proposed method to reduced partial views of the liver. For this experiment, there is a rigid transformation between the moving (*M*) and target (*T*) models, with all the remaining parameters fixed.

In this experiment, the target model, *T*, is simulated by creating 10 partial views of decreasing sizes (from 43 to 7%) by cropping the original liver mesh *M*. This step was manually performed in Meshlab. Each algorithm is run 500 times for each size. The mean and standard deviation of the resulting errors are reported in Fig. 3-top.

#### Robustness to deformation

We validate the robustness of the proposed algorithm to increasing levels of deformation in the data. A large deformation is applied with control points on the left lobe of the liver mesh *M*. Intermediate levels of deformation are generated with vertex linear morphing between the original liver shape and the deformed one. These steps were achieved in MeshMixer and Meshlab. Figure 3-bottom shows examples of the different deformation levels with 17 being the highest.

We choose the size of the partial view as 23% (Fig. 3-top, middle shape) since this can be registered well by all algorithms tested. Visually, it represents a realistic size for a laparoscopic view.

In this experiment, the deformation level is the only variable. Each algorithm is run 500 times for each deformation level. The mean and standard deviation are presented in Fig. 3-bottom.

### Liver phantom

The proposed method is validated using the OpenCAS [8] public dataset, which contains 3D meshes from an experiment in which a silicone liver phantom is deformed by an indentation. The positions of small Teflon marker balls in both the initial and deformed states of the phantom are given. Please refer to Suwelack et al. [8] for more details about how the dataset was built.

The 3D model of the liver phantom in its initial state is used as the moving model, *M*. The proposed coarse registration method is tested in two scenarios. Firstly, a partial view of the deformed liver phantom is used as the target model, *T* (Fig. 4-left), which tests the performance under deformation, partial and sparse data. Secondly, a partial surface reconstructed from an intraoperative stereo endoscopic camera (Fig. 4-right) is used as *T*. On top of deformation and partial data, this scenario also tests the proposed method in realistic noise levels from a stereo reconstruction. After the global alignment is estimated with the proposed method, Levenberg–Marquardt iterative closest point (LM-ICP)[10] is applied. The distribution of target registration error (TRE) in mm is computed for both cases. The mean TRE for the partial deformed surface is 7.94 mm after our proposed global alignment, which is further reduced to 7.77 mm after LM-ICP. Similarly, in the case of the intraoperative partial surface, the mean TRE of 28.62 mm after the global alignment is decreased to 12.10 mm after LM-ICP. The best case scenario for rigid registration would be point-based alignment of the marker ball positions before and after deformation, with a mean TRE of 5.66 mm.

### Application in clinical data

The proposed approach is demonstrated on clinical data from a video sequence acquired during a laparoscopic liver resection. The 3D mesh of the liver surface was extracted from a CT scan before surgery.^2^ We use the SmartLiver system [5] to process the retrospective data. The liver is automatically segmented in the laparoscopic video with a deep learning framework [30]. Surface patches are collected to cover all the visible surface in each video [31]. They are consequently merged together using optical tracking data.

**Fig. 5:**
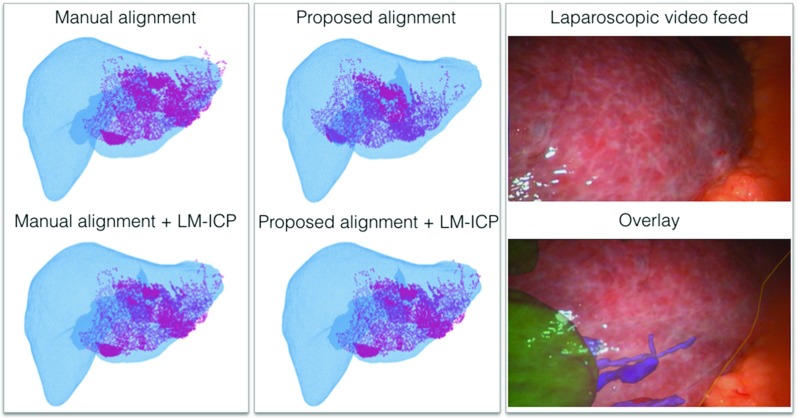
Global alignment on clinical data from a dataset acquired during a laparoscopic liver resection. *Color coding Alignment:* moving model, *M*—blue, target model, *T*—pink. The overlay is computed after applying LM-ICP on the proposed alignment. *Color coding Overlay:* liver tumour—green, vessels—purple, liver contour—yellow

Figure 5 shows the visual assessment between the manual alignment performed on the SmartLiver GUI and the proposed method. The last column illustrates an example of augmented reality in laparoscopic liver surgery after LM-ICP is applied to the proposed alignment.

## Discussion

The results from the first experiment show that when the intraoperative surface is large enough, all three methods have comparable results. However, having surfaces with size smaller than 23% of the whole mesh becomes challenging for both *R* and $SM+R1$. From what we noticed in our datasets, such surfaces are characteristic for videos acquired on the left lobe in laparoscopic interventions with restricted camera movement. Note how the proposed method has less variance in the solutions even for smaller partial shapes. The additional pairwise term which incorporates the boundaries of both *M* and *T* makes the problem less ambiguous, as opposed to just using the geodesic distances between pairs of correspondences.

The second experiment illustrates robustness to increasing deformation levels in the partial views. Similarly to the previous experiment, the proposed method is more consistent across different deformations, with less variation in the solutions it provides. This is mostly due to the fact that the set of correspondences, $C_\mathrm{p}$, obtained from the proposed method contains fewer outliers than the other methods.

These methods are compared with RANSAC because it is a popular algorithm for finding correspondences between point sets related by parametric transformations. If RANSAC is applied directly on the set of correspondences *C*, it is unable to obtain a good alignment. This is mostly due to the fact that the initial set will contain a high number of outliers, due to the low descriptiveness of the data. Moreover, for partial shape sizes characteristic to laparoscopic surgeries (less than 23% in Fig. 3), there are multiple locations on the liver which result in a good fit. However, by allowing for deformation in the proposed pruning technique, a set of correspondences, $C_\mathrm{p}$, is obtained in the correct region of interest, which can be further refined by RANSAC. This approach is also more computationally efficient since RANSAC has to find three good pairs of correspondences from small sets (approximately 10 correspondences, depending on the data).

A quantitative evaluation of the proposed algorithm is performed on a phantom dataset with partial size, deformation and realistic noise levels. The partial surface used in Fig. 4-right is illustrative for an intraoperative scenario, since the data are collected using a stereo laparoscope. The proposed method succeeds in providing a good initial alignment, and it is shown that further fine alignment methods (such as LM-ICP) can successfully converge towards the correct location. The current errors are comparable to the literature in the rigid case scenario [5–7]. Since most fine registration algorithms can converge successfully if the coarse alignment is within a few cm [9], the proposed method achieves results within the desired range. In order to decrease the errors further, we will investigate non-rigid refinement methods in the future.

We show promising results on a retrospective video acquisition from a laparoscopic liver surgery. The proposed method is compared against a manual alignment performed on the SmartLiver GUI. Qualitative results are provided to illustrate that the proposed method manages to correctly identify the liver region in a challenging environment with realistic noise levels, significant deformation and small partial views. Note that the proposed method aims to estimate a coarse surface alignment, which can then be further refined with a local algorithm. For example, Fig. 5-right shows an overlay computed by applying LM-ICP on the coarse alignment estimated by our method. Furthermore, the current computational time required to compute the initial alignment between surfaces is approximately 20 s with non-optimised code, which makes it feasible for clinical usage.

Our experiments were all performed using the same choice of parameters, suggesting the proposed method is not very sensitive to variations. However, in the future we would like to investigate their influence. Although the experiments presented here show promising results, we would like to validate the proposed technique on clinical data from more patients to test its robustness with respect to liver surface variations. Furthermore, we are looking into ways to automate the liver contour selection, such that no user interaction is needed and the whole process is fully automated.

## Conclusion

We propose a fast and global method for surface-based registration of a 3D liver mesh extracted from a preoperative CT scan of the liver and the surface reconstruction of the intraoperative laparoscopic video feed. We have validated its performance with respect to the challenges characteristic to laparoscopic surfaces on synthetic data, on a phantom dataset and on retrospective clinical data. We conclude that the proposed method could potentially be used as an automatic way of obtaining a good initial alignment between the two surfaces, given the required features. Moreover, it does not require any advanced hardware, which makes it accessible and comparatively easy to translate to a clinical setting.

## Footnotes

1 http://www.meshmixer.com.

2 www.visiblepatient.com.
